# Assessment of *Plasmodium falciparum* PfMDR1 transport rates using Fluo-4

**DOI:** 10.1111/jcmm.12313

**Published:** 2014-06-01

**Authors:** O Friedrich, SJ Reiling, J Wunderlich, P Rohrbach

**Affiliations:** a Institute of Medical Biotechnology, Friedrich-Alexander-University Erlangen-NurembergErlangen, Germany; b SAOT, Erlangen Graduate School of Advanced Optical Technologies, Friedrich-Alexander-University Erlangen-NurembergErlangen, Germany; c Institute of Parasitology, McGill UniversitySte. Anne de Bellevue (Montreal), QC, Canada

**Keywords:** *Plasmodium falciparum*, PfMDR1, transport kinetics, Fluo-4, live-cell imaging, calcium, pH

## Abstract

Mutations in the multidrug resistance transporter of *Plasmodium falciparum* PfMDR1 have been implicated to play a significant role in the emergence of worldwide drug resistance, yet the molecular and biochemical mechanisms of this transporter are not well understood. Although it is generally accepted that drug resistance in *P. falciparum* is partly associated with PfMDR1 transport activity situated in the membrane of the digestive vacuole, direct estimates of the pump rate of this transport process in the natural environment of the intact host–parasite system have never been analysed. The fluorochrome Fluo-4 is a well-documented surrogate substrate of PfMDR1 and has been found to accumulate by actively being transported into the digestive vacuole of several parasitic strains. In the present study, we designed an approach to use Fluo-4 fluorescence uptake as a measure of compartmental Fluo-4 concentration accumulation in the different compartments of the host–parasite system. We performed a ‘reverse Fluo-4 imaging' approach to relate fluorescence intensity to changes in dye concentration rather than Ca^2+^ fluctuations and were able to calculate the overall rate of transport for PfMDR1 in Dd2 parasites. With this assay, we provide a powerful method to selectively measure the effect of PfMDR1 mutations on substrate transport kinetics. This will be of high significance for future compound screening to test for new drugs in resistant *P. falciparum* strains.

## Introduction

Malaria remains a major health problem in many parts of the world, particularly sub-Saharan Africa, where ∼216 million cases of infection and 665,000 to 1.2 million deaths occur annually [1,2]. The emergence and spread of multidrug-resistant *Plasmodium falciparum* parasites have severely compromised the ongoing global efforts to control the disease [3].

P-glycoprotein transporters are often implicated in disease aetiology and treatment. Investigations into *P. falciparum* resistance have included *pfmdr1*, a parasite homologue of the mammalian multidrug resistance ATP binding cassette (ABC) transporter family. *Pfmdr1* encodes a 162 kD protein that consists of two homologous parts, each comprising a cytosolic nucleotide-binding domain (NBD) and a substrate-binding transmembrane domain (TMD) with 12 putative transmembrane regions.

In the malaria parasite, PfMDR1 has been localized to the membrane of the parasite's digestive vacuole (DV) [4], an acidic lysosome-like organelle [5], and has been shown to transport solutes into the DV [6]. If similar to MDR1 transporters in the plasma membrane of mammalian cells, PfMDR1 is anticipated to bind its substrates in the cytosolic membrane leaflet of the DV membrane and flip them to the inner DV leaflet at the expense of ATP hydrolysis.

PfMDR1, as all MDR1 transporters, has broad substrate specificity, enabling the transport of a diverse array of substances. In the past, fluorochromes have been used in a wide range of assays to assess P-gp function [7]. Recently, we showed that the fluorochrome Fluo-4 is a substrate for PfMDR1. Fluo-4 is commonly used as a non-ratiometric Ca^2+^ indicator in eukaryotic cells but has also been used to evaluate multidrug transporter activity in lymphocytes [8]. In *P. falciparum*, Fluo-4 provided evidence for active transport of the dye into the parasite's DV [6]. Furthermore, the mutant *pfmdr1* gene (Y86, Y184, S1034, N1042, D1246) encodes for a protein that is more effective in transporting Fluo-4 from the cytosol of the parasite into the DV than the wild-type PfMDR1 protein (N86, F184, S1034, D1042, D1246) [6]. Although these studies showed that, under steady-state conditions, the accumulated staining pattern of Fluo-4 fluorescence in the DV was a marker to differentiate between drug-sensitive *versus* -resistant strains [6], the kinetics of this transport process reflecting the elementary mode of action of this drug resistance transporter remained elusive.

**Fig. 1:**
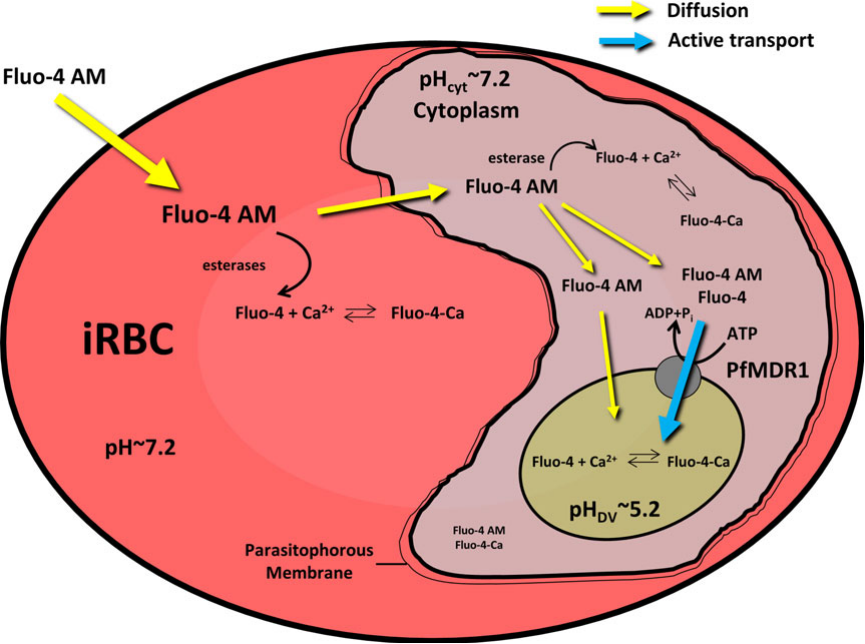
Proposed model of Fluo-4 distribution within the compartments of *Plasmodium falciparum* (Dd2)-infected erythrocytes. Bulk [Fluo-4]-AM is taken up into the erythrocyte cytosol by passive diffusion, where the acetoxymethyl-ester is partially cleaved by esterases, making it membrane impermeant. Uncleaved Fluo-4 AM is being redirected into the parasite's cytoplasm through the PV membrane, where it is cleaved and slowly accumulates in this compartment. In the Dd2 parasite, transport of Fluo-4 into the digestive vacuole occurs both *via* diffusion (Fluo-4 AM) and by active transport *via* the PfMDR1 pump, which transports both the AM and the cleaved Fluo-4 dye.

Understanding the bioenergetics of PfMDR1 sheds light on general mechanisms of action for this superfamily of transporters. Unlike artificial systems, such as PfMDR1 embedded in artificial liposomes, it is desirable to study transport rates in the natural environment of the transporter, *i.e*. within the DV of the intact *P. falciparum*-infected erythrocyte and to have tools at hand to differentiate between pump rates and diffusional uptake. Therefore, the aim of the present study was to investigate the transport kinetics of PfMDR1 in the host–parasite system using the Fluo-4 fluorochrome and live-cell imaging. We describe a detailed investigation of the transporter in relation to Fluo-4 solute uptake applied in all compartments of the host–parasite system (Fig. 1). This is the first estimate of overall transport rates of PfMDR1 in the intact host–parasite system. Using a ligand-binding model, we were able to estimate a maximum overall pump rate of ∼50,000 Fluo-4 molecules/min.—or 820/sec.—for the complete set of PfMDR1 molecules found on the DV membrane of the Dd2 parasite.

## Materials and methods

### Cultivation of *P. falciparum parasites*

*Plasmodium falciparum blood stage* parasites (HB3, Dd2) were maintained in culture using a modified protocol of Trager and Jensen [9]. Parasites were propagated using human A^+^ erythrocytes in complete RPMI medium supplemented with 0.5% AlbuMAX II (Life Technologies Inc., Burlington, ON, Canada) at 37°C in an atmosphere of 92% N_2_, 3% O_2_, 5% CO_2_. All experiments were carried out using trophozoite stage parasites (28–36 hrs post invasion).

### Dye loading of *P. falciparum* parasites

Trophozoite stage *P. falciparum*-infected erythrocytes (28–36 hrs post invasion) were washed twice with Ringer's solution (122.5 mM NaCl, 5.4 mM KCl, 1.2 mM CaCl_2_, 0.8 mM MgCl_2_, 11 mM D-glucose, 10 mM HEPES, 1 mM NaH_2_PO_4_, pH 7.4) and loaded with 0.1–15 μM Fluo-4-AM (Life Technologies Inc.) in Ringer's solution with Pluronic F-127 (0.1% v/v) for 0–180 min. at 37°C, depending on the experiment. Since all parasites are cultured in RPMI media supplemented with Albumax (*i.e*. no serum present), influences of extracellular serum esterases on Fluo-4 loading of cells as described in [10] are of no concern here. Dye-loaded parasites were settled onto poly-l-lysine-coated coverslips in a micro-perfusion chamber as described in [11]. Unbound parasites and remaining dye were washed away by perfusion with Ringer's solution.

### Live-cell imaging

Confocal laser scanning fluorescence microscopy was performed using a Zeiss LSM710 (Carl Zeiss, Jena, Germany) equipped with visible laser lines and an Axiovert 200M microscope. Fluo-4 was excited at 488 nm with 1% transmission (argon laser, 25 mW), and emission signals were detected in the range of 505–560 nm. Single images were obtained using a 63× objective (C-APO, N.A. = 1.2) and a fivefold software zoom, 512 × 512 pixel. Fluo-4 fluorescence was assessed using *P. falciparum*-infected erythrocytes incubated with Fluo-4 AM for various times (0–180 min.). Images of 20–30 parasites were taken every 30 min. and repeated in three subsequent experiments on varying days. This single image snapshot approach of quantifying numerous parasites at a given time was chosen over a long-time course of Fluo-4 fluorescence recording to quantify one individual parasite because the DV is known to be very light sensitive in response to repetitive illumination recordings [12,13]. Our approach minimizes artefacts that may be caused through DV photolysis when imaging a single parasite for more than a few minutes. Regions of interest (ROI) within the infected erythrocytes were identified and fluorescence intensities quantified in the respective compartments, *i.e*. erythrocyte cytosol, parasite cytoplasm and DV. Mean values and standard errors were computed for each strain and each incubation time-point. The mean time course of rise in Fluo-4 fluorescence in each compartment stems from the rise in total Fluo-4 concentration ([Fluo-4]_tot_) according to the equation


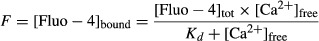
(1)

because intracellular [Ca^2+^] does not change during loading conditions [6,12]. As Fluo-4 fluorescence, F, increases over time during the loading period, the time course of F can serve to calculate [Fluo-4]_tot_(*t*) for each compartment. In particular, the overall pump rate for Fluo-4 into the DV *via* all PfMDR1 proteins active on the DV membrane can be obtained by comparing HB3 and Dd2 strain behaviour according to a multi-compartment model presented in Figure 1. [Fluo-4]_tot_(*t*) can be calculated from F if the dissociation constant (*K*_*d*_) of the Fluo-4–Ca^2+^ buffer reaction is known for the respective compartment (*i.e*. the true pH environment) and the steady-state free Ca^2+^ ([Ca^2+^]_free_) was given. The time course of fluorescence, F(*t*), in the respective compartments was normalized to the initial background condition, F(0), at time-point zero (immediately after adding Fluo-4), to compare relative fluorescence between strains and batches and to avoid artefacts from fluctuating background conditions. For the time course of normalized F(*t*), the equilibrium described by Eq. (1) is still influenced by differences in compartmental pH (*i.e*. ∼7.2 in the parasite cytoplasm and ∼5.2 in the DV). pH effects on F(*t*) cannot be calculated from Eq. (1). Therefore, to be able to relate relative (normalized) fluorescence F(*t*) to steady-state fluorescence for a given total [Fluo-4]_tot_ and [Ca^2+^]_free_ at a given pH, an *in vitro* calibration was performed as described below, which served as input to convert F(*t*) values from the live-cell recordings to [Fluo-4]_tot_ concentrations. This conversion enabled us to calculate kinetics of absolute Fluo-4 dye accumulation characteristics in the compartments *in situ*.

### Quantification of apparent *K*_*d*_ of Fluo-4

To compute the apparent *K*_*d*_ for Fluo-4 using the Fluo-4-Ca^2+^ buffer reaction in the host–parasite system, a confocal laser scanning microscope (Carl Zeiss) was used to perform *in situ* Ca^2+^ calibration. Parasites were loaded with 5 μM Fluo-4-AM at 37°C (in Ringer's solution) and added to a chamber as described above. The mean Fluo-4 fluorescence intensities of the various compartments were evaluated after permeabilizing the membranes with the Ca^2+^ ionophore ionomycin (10 μM) and clamping the external solution to free Ca^2+^ values of 1 nM, 150 nM, 351 nM, 602 nM, 39 μM and 1.2 mM. A calibration curve was fitted to the Ca^2+^–F_Fluo-4_ relationship using a least square fit to a Hill function. Fluorescence values were normalized to the basal fluorescence intensity at pCa 9 and the apparent *K*_*d*_ values for each compartment (*i.e*. RBC cytosol, iRBC cytosol, parasite cytoplasm, DV) were determined from the sigmoidal curves.

### *In vitro* calibration of Fluo-4 fluorescence at pH 5.2 and 7.2

To convert normalized Fluo-4 fluorescence, F/F(0), in the compartments to absolute dye concentrations at any given time, and to account for the pH differences affecting the dye buffer properties (and thus, fluorescence values), an *in vitro* calibration of Fluo-4 fluorescence in intracellular solutions containing a fixed concentration of total Fluo-4 (0, 0.1, 0.4, 1, 5, 15, 50 or 100 μM) and various free [Ca^2+^] values was performed. Intracellular solutions were based on ‘high Ca^2+^’ and ‘Ca^2+^ free’-solutions (termed HA and HR, respectively). HA consists of 30 mM Hepes, 30 mM EGTA, 30 mM CaCO_3_, 7.46 mM Mg(OH)_2_, 10 mM Na_2_-creatine phosphate, 8 mM Na_2_ATP; HR is the same as HA except with 8.1 mM Mg(OH)_2_ and omitting CaCO_3_. The pH was adjusted either to 5.2 or 7.2 using KOH or HCl. Mixtures of HA:HR were pipetted at ratios ranging between 0 and 0.9 in 96-well plates in a matrix, where rows represented HA:HR ratios and columns were supplemented with the respective Fluo-4 salt concentrations. Plates were prepared at least in duplicates for pH 5.2 and pH 7.2, respectively. After ∼30 min. incubation on a rocker shaker, Fluo-4 fluorescence in each well was assessed using a Synergy H4 plate reader (Bio-Tek Instruments Inc., Winooski, VT, USA). Each well corresponded to a Fluo-4 value at a given pH, [Fluo-4]_tot_ and total [Ca^2+^]. To convert total [Ca^2+^], ([Ca^2+^]_tot_) (which was the same irrespective of pH) to free [Ca^2+^] concentrations [Ca^2+^]_free_ (which can deviate significantly at different pH values), the chelating program React II was used (developed by Godfrey Smith, University of Glasgow). This program calculates [Ca^2+^]_free_ from known solution compositions of Ca^2+^-buffered solutions with known [Ca^2+^]_tot_ and pH using published critical stability constants. Using this conversion, Fluo-4 fluorescence values at a given pH and [Ca^2+^]_free_, normalized to pCa 9 (background), could be compared with the normalized F(*t*) values from the live-cell *P. falciparum*-infected erythrocyte recordings to calculate absolute [Fluo-4]_tot_ concentrations, after establishing calibration curves for each [Fluo-4]_tot_ concentration over the complete [Ca^2+^]_free_ range.

### Image analysis

*P. falciparum*-infected erythrocytes were readily identified by eye. Using the Zen software (Carl Zeiss), a region of interest within the host–parasite system, *e.g*. the DV, the cytoplasm of the parasite, the cytosol of the infected RBC or the uninfected RBC, was defined. The average pixel intensity of the region of interest was calculated. The measured fluorescence was further analysed using Excel (Microsoft Canada Corp., Quebec, QC, Canada), Sigma Plot (SPSS Inc., Chicago, IL, USA), and ImageJ 1.47g (National Institutes of Health, Bethesda, MD, USA). Fluorescence values were normalized to the initial condition, *i.e*. time-point zero after Fluo-4 incubation, F(*t*) = F/F(0), for *in situ* time series of Fluo-4 uptake. Images were processed using Corel Draw and Corel Photopaint (Corel Corp., Ottawa, ON, Canada).

### *pfmdr1* sequence analysis

The full length sequence of *pfmdr1* was verified in our laboratory HB3 and Dd2 strains. Parasite strains were grown to ≥5% parasitaemia and DNA extracted using the QIAamp DNA Blood Mini Kit (Qiagen Inc., Toronto, ON, Canada) according to the manufacturer's instructions. The DNA was amplified in overlapping PCR fractions using HotStarTaq DNA polymerase (Qiagen Inc.). To account for the AT-rich nucleotide content in the *P. falciparum* genome, dNTPs (Invitrogen Canada Inc., Burlington, ON, Canada) were mixed at 75% AT and 25% GC. For PCR optimization, 2 mM MgCl_2_, 300 μM dNTPs and 300 nM primers were used for the reaction. For each reaction mix, a total of 20 ng genomic DNA was used. PCR reactions consisted of an initial activation step of 94°C for 3 min. followed by 35 cycles of 94°C for 60 sec., 49–61°C (adjusted for each primer pair) for 30 sec. and 72°C for 1 min. Primers used for *pfmdr1* gene sequencing were: *pfmdr1.1_F:* 5′-AT ATATGTGTACATAGCTTATTTCA, *pfmdr1.1_R:* 5′-TTGTACTAAACCTATAGA TACTAATGA, *pfmdr1.2_F*: 5′-GTTTAAATGTTTACCTGCACAACATAGAAAA, *pfmdr1.2_R:* 5′-CTCCACAATAACTTGCAACAGTTCTTA, *pfmdr1.3_F*: 5′-ATGCACGTTTGACTTTATGTATTAC, *pfmdr1.3_R:* 5′-GGATCTTGACTAACAACTCCAA, *pfmdr1.4_F:* 5′-CTTTATGATCCAACCGAAGGAGATA, *pfmdr1.4_R:* 5′-GTTATCCGATCCATTATCATTTCCA, *pfmdr1.5_F:* 5′-ACAAGGTACACATGATAGTCTTATGA, *pfmdr1.5_R:* 5′-GGATTTCATAAAGTCATCAACTAATATAG, *pfmdr1.6_F*: 5′-CTGTAATTTGATAGAAAAAGCTATTGATTA, *pfmdr1.6_R:* 5′-CCATATGGTCCAACATTTGTATC, *pfmdr1.7_F:* 5′-CAATAGTTAGTCAAGAACCCATGTTA, *pfmdr1.7_R:* 5′-TCAATATAACGGACAAGAGTTGATAC, *pfmdr1.7.1_F:* 5′-ACCAATCTGGATCTGCAGAAGA, *pfmdr1.7.1_R:* 5′-TCTCAGAATTGGAATCAAGTGATGA. If DNA amplification using *pfmdr1.7* primers was unsuccessful, the PCR was repeated with the alternative primers *pfmdr1.7.1*. Samples were sent for sequencing to Genome Quebec, Canada and analysed using the BioEdit software [14].

### Real-time PCR

*pfmdr1* gene copy numbers were determined through real-time PCR using fluorescent TaqMan probe-based gene expression. Primer pairs of *pfmdr1* [15] and the housekeeping gene *seryl-t-rna-synthetase* were designed to match in length and nucleotide content of the two amplified gene regions. In addition, TaqMan probes with a FAM or VIC dye on the 5′ end and a TAMRA quencher on the 3′ end were added to the reaction mix (Applied Biosystems, USA). The primers used for quantifying copy number were: *PF-F:* 5′-TTAAGTTTTACTCTAAAAGAAGGGAAAACATA, *PF-R:* 5′-TCTCCTTCGGTTGGATCATAAAG, *PF-FAM:* 5′-FAM-CATTTGTGGGAGAATCAGGTTGTGGGAAAT-TAMRA, seryl_F:5′-GATTTATTAAGAAAAATAGGTGGAGCTA, seryl_R: 5′-TATAGCATTATGTAATAAGAAACCTGC, seryl_probe: 5′ VIC-AAGGTATACAAGTAGCAGGTCATCGTGGTT-TAMRA. The reaction mix contained 20 ng DNA, 300 nM primers, 250 nM TaqMan probes, 300 μM dNTPs, 2 mM MgCl_2_ and 2.5 units/reaction HotStarTaq DNA Polymerase (Qiagen). Samples were prepared in triplicates. For the reaction, the initial activation step was at 94°C for 3 min., followed by 40 cycles of 94°C for 1 min., 60°C for 1 min. and 72°C for 30 sec. Fluorescence was recorded after each elongation step. Real-time PCR was carried out in a Rotor-Gene RG-3000 (Corbett Research, Toronto, ON, Canada). Copy numbers were determined relative to 3D7, which is known to have only one *pfmdr1* gene copy [16,17].

## Results

### Fluo-4 fluorescence accumulation in *P. falciparum*-infected erythrocytes

In a previous study, we showed that the fluorochrome Fluo-4 is actively transported into the DV of chloroquine-resistant (CQR) Dd2 but not chloroquine-sensitive (CQS) HB3 parasites *via* the multidrug resistance transporter PfMDR1 [6]. In the present study, we have extended this approach on live cells using Fluo-4 fluorescence detection in *P. falciparum*-infected erythrocytes as an *in situ* assay to directly monitor vacuolar PfMDR1 transport and establish the overall pump rate of this transporter in Dd2 parasites. This is possible as the increase in Fluo-4 fluorescence in the DV is related to the dye accumulation in this compartment *via* diffusion and PfMDR1 transport activity (Fig. 1). PfMDR1 activity can thus be dissected out comparing group data from HB3 strains (pure diffusion model) and Dd2 strains (diffusion and active pump model).

**Fig. 2:**
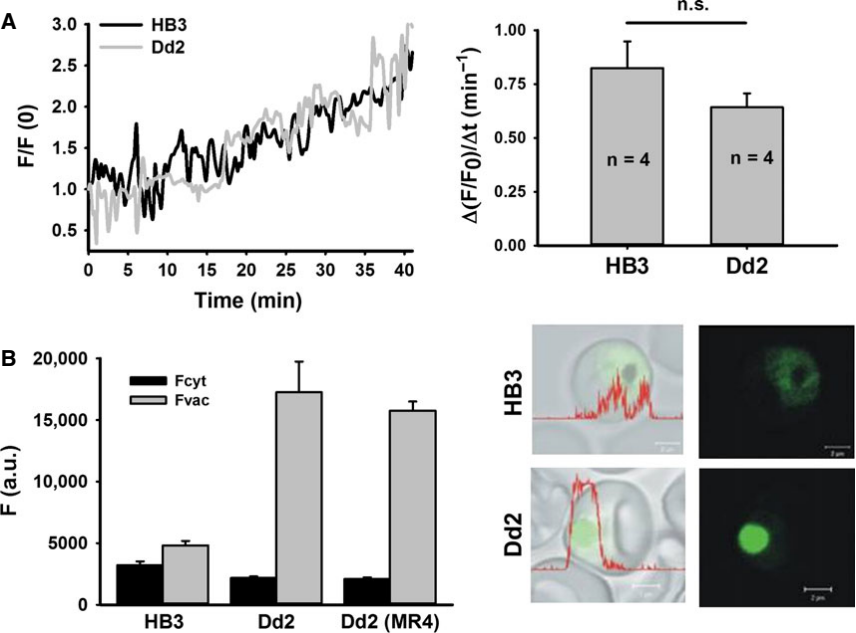
Global Fluo-4 uptake and fluorescence in HB3 and Dd2 *Plasmodium falciparum*-infected erythrocytes. (**A**) A time-lapse series measured the increase in fluorescence intensity within the complete host–parasite system circumference during incubation with 5 μM Fluo-4 AM in the external bulk solution. During this time frame (40 min), HB3 and Dd2 parasites showed a linear global Fluo-4 fluorescence increase and revealed similar uptake rates when all compartments were merged (left panel). The calculated increase rate of global fluorescence was not significantly different between HB3 and Dd2 parasites (*n* = 4 per strain, *P* = 0.24; right panel). (**B**) Mean Fluo-4 fluorescence quantified from the digestive vacuolar and cytoplasmic regions of several *P. falciparum* parasites. Dd2 parasites from our laboratory and Dd2 parasites from MR4 showed no significant difference in fluorescence. The mean ± SEM of at least 17 independent determinations collected over several days is shown (left). The right panel shows single images of *P. falciparum*-infected erythrocytes stained with Fluo-4 AM (green); scale bar, 2 μm.

To determine live *in situ* Fluo-4 uptake rates in the intact host–parasite system, it is essential to compare the global fluorescence increase representing the Fluo-4 uptake within CQS and CQR parasites to verify for possible differences in global uptake kinetics (*i.e*. all compartments merged). Evaluation of total global Fluo-4 fluorescence signals within the intact HB3 or Dd2 *P. falciparum*-infected erythrocyte (comprising all compartments of the host–parasite system, including the erythrocyte cytosol, parasite cytoplasm and DV) revealed no significant difference in fluorochrome uptake kinetics within the 40 min. time interval investigated (Fig. 2A). The temporal profiles of the integrated fluorescence intensity show a complete overlap for both strains, suggesting a linear increase in global Fluo-4 accumulation. However, when the fluorescence signals within the respective single compartments of the host–parasite system were investigated (*i.e*. erythrocyte cytosol, parasite cytoplasm, DV), Fluo-4 accumulation varied substantially among the compartments of the two strains. While HB3 parasites generally showed a uniform Fluo-4 accumulation throughout the whole parasite (difference not significant), Dd2 parasites displayed an increased accumulation of Fluo-4 signal in the DV (Fig. 2B). This prompted us to focus on the time resolved quantification of Fluo-4 fluorescence in each separate compartment.

Our previous work showed that the accumulation of Fluo-4 in the DV of Dd2 parasites is mainly due to the pump action of PfMDR1, which is modulated through mutations in this transporter [6]. HB3 parasites do not transport Fluo-4 into the DV and take up this dye through passive diffusion (Fig. 1). To verify mutations that facilitate Fluo-4 transport, we sequenced the complete *pfmdr1* gene for both HB3 and Dd2 parasites cultured in our laboratory in addition to determining the gene copy numbers of these strains. We confirmed that only three PfMDR1 polymorphisms at amino acid positions 86, 184 and 1042 differed between the two parasites (Table 1). Interestingly, the PfMDR1 amino acid sequence of Dd2 revealed both tyrosine (Y) and phenylalanine (F) at position 86. A serial dilution of this strain was carried out and re-sequencing confirmed the Y/F mutation. *Pfmdr1* copy numbers for HB3 and Dd2 parasites were verified and calculated to be 1 and 2, respectively. Because of amino acid alterations seen in our Dd2 strain, an MR4-obtained Dd2 strain (MRA-150, MR4, ATCC®, Manassas, VA, USA) was sequenced to confirm the amino acid variation seen at position 86. The *pfmdr1* copy number of this MR4 strain was found to be 3 (Table 1). For this study, we used the Dd2 strain containing two copies of *pfmdr1*.

**Table 1:** PfMDR1 amino acid sequences and copy numbers for strains used in this study

	PfMDR1 amino acid sequence					*pfmdr1*
		
	86	184	1034	1042	1246	copy number
HB3	N	F	S	D	D	1
Dd2 (our laboratory)	F/Y	Y	S	N	D	2
Dd2 (MR4)	F/F/Y	Y	S	N	D	3

**Fig. 3:**
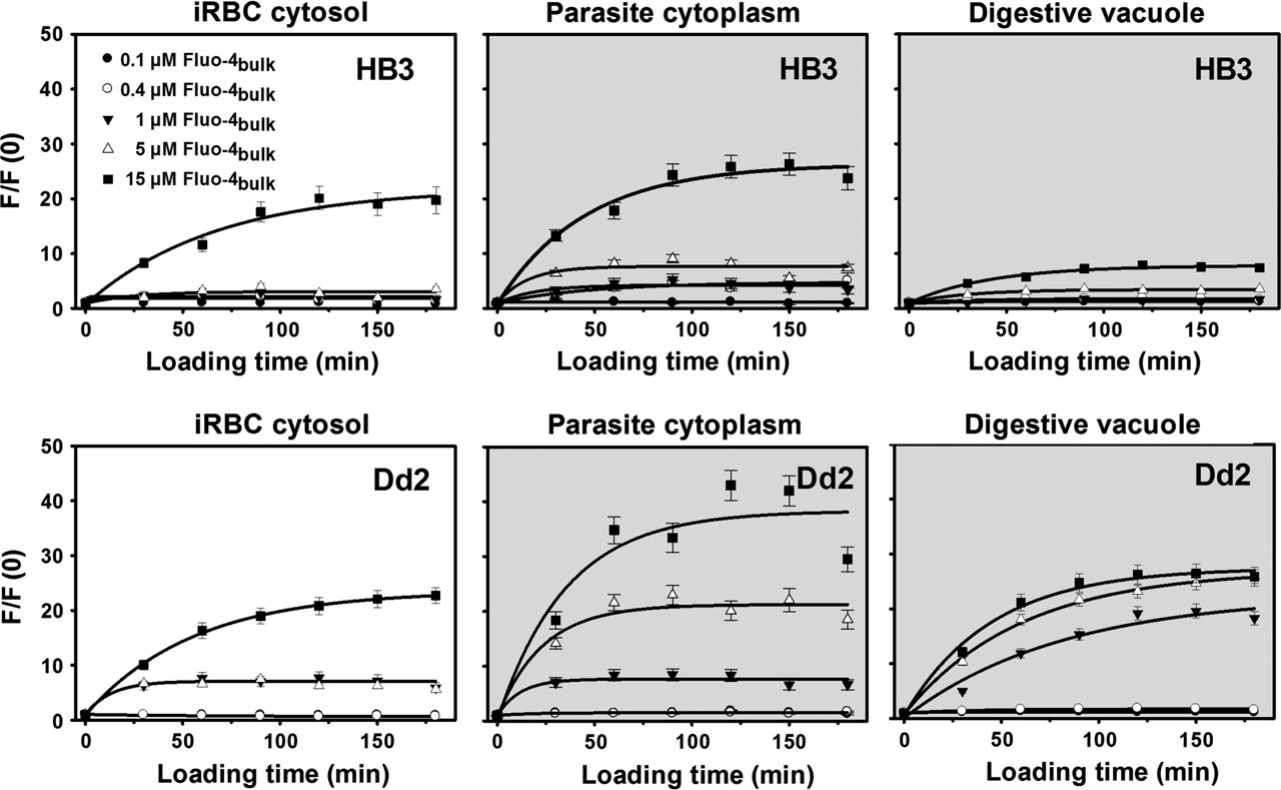
Fluo-4 fluorescence increase over time. Time course of Fluo-4 fluorescence increase in the different compartments of HB3- and Dd2-infected erythrocytes for loading times between 0 and 180 min. with different bulk Fluo-4 AM concentrations indicated. Values at each time-point are obtained from at least 30 individual parasites. Fluorescence was normalized to time-point zero, F/F(0). An exponential fit was derived for each bulk concentration.

To deduce kinetics properties for PfMDR1, one must follow the time course of Fluo-4 fluorescence (F_Fluo-4_) accumulation in the host–parasite system. To evaluate F_Fluo-4_ increases in the various host–parasite compartments over time, we measured F_Fluo-4_ within this system over 3 hrs using five different external Fluo-4 AM concentrations in the bulk solution (0.1–15 μM). For both HB3- and Dd2-intact multi-compartment systems, the time course of F/F(0) (normalized F_Fluo-4_) during dye loading was quantified for the iRBC cytosol, the parasite cytoplasm and the parasite's DV (Fig. 3). The normalized fluorescence F(*t*) = F/F(0) of Fluo-4 dye loading was found to be comparable in the iRBC and parasite cytoplasm compartments of HB3 and Dd2 parasites but differed significantly for the DV.

### Apparent *K*_*d*_ of Fluo-4

**Fig. 4:**
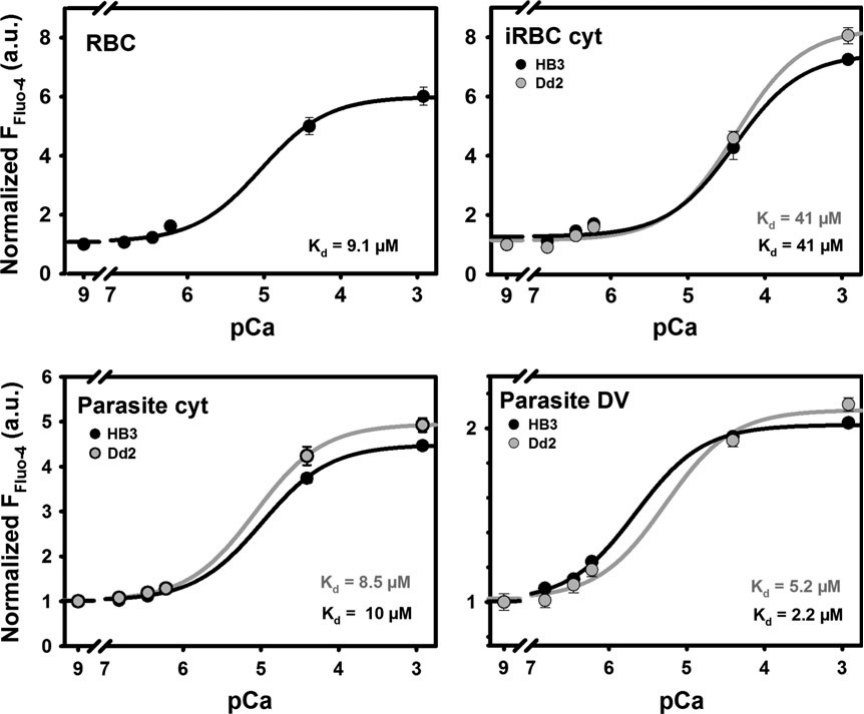
Normalized pCa–Fluo-4 steady-state fluorescence relations. *In situ* calibration relationships were applied to obtain apparent *K*_*d*_ values in the different compartments of HB3- and Dd2-infected erythrocytes. Steady-state Fluo-4 fluorescence intensities are given for different clamped pCa values in ionomycin-permeabilized HB3- or Dd2-infected erythrocytes (iRBC) or non-infected erythrocytes (RBC). Within the parasites, the cytoplasmic and DV compartments were evaluated. Fluorescence values were normalized to the basal fluorescence intensity at pCa 9 (essentially no Ca^2+^ present) and the apparent *K*_*d*_ values extracted from the sigmoidal relationships. External Fluo-4 AM concentration was 5 μM. The iRBC represents a low-affinity compartment compared to RBC cytosol, most likely due to the drain of Ca^2+^ into the parasitic compartments. From the reconstructed calibration curves, the apparent Fluo-4 fluorescence derived compartmental Ca^2+^ concentrations were obtained (see Table 2).

As Fluo-4 only fluoresces when bound to free Ca^2+^, we sought out to quantify the *K*_*d*_ for the reaction of this dye in each compartment of the parasite. The increase in fluorescence intensity with increasing [Ca^2+^] was used in an *in situ* calibration procedure to obtain the apparent steady-state *K*_*d*_ of the dye. To calibrate F_Fluo-4_ to [Ca^2+^]_free_ in each compartment, the normalized steady-state fluorescence intensity F/F(0 Ca^2+^) (normalized to base intensities at diminished Ca^2+^ levels of pCa ∼9) was measured (*n* > 20 parasites). Figure 4 shows the calibration results in the host erythrocyte compartments of uninfected (RBC) and HB3- or Dd2-infected erythrocytes (iRBC) as well as the parasitic compartments. Using the fit-derived *K*_*d*_ values, the apparent steady-state Ca^2+^ levels for each compartment were determined from resting F_Fluo-4_ values as given in Table 2. It is important to note that the right-shifted pCa–Fluo-4 plot for the DV of Dd2 parasites in Figure 4 suggests a weaker binding affinity of Fluo-4 to Ca^2+^ in this compartment when compared to HB3 parasites.

**Table 2:** Comparison of Fura-Red derived resting [Ca^2+^] in the different compartments of erythrocyte cytosol (RBC_cyt_, iRBC_cyt_), parasite cytoplasm (Par_cyt_), and digestive vacuole (Par_vac_) with the apparent Fluo-4 derived Ca^2+^ concentrations using the *in situ* calibration results and resting Fluo-4 fluorescence intensities from hundreds of intact infected and non-infected erythrocytes. The non-ratiometric dye Fluo-4 suggests an inaccurately elevated [Ca^2+^] in the parasitic compartments of Dd2, as the concentration of dye was incorrectly assumed to be evenly distributed. In contrast, the ratiometric dye Fura-Red indicates no major Ca^2+^ storage properties of the digestive vacuole

Compartment	Fura-Red [Ca^2+^]_resting_ (μM) [12]	Fluo-4 *K*_*d*_ (μM)	Apparent Fluo-4 [Ca^2+^]_resting_ (μM)
RBC			
RBC_cyt_	0.16	9.10	1.40
HB3			
iRBC_cyt_	0.08	41.00	2.30
Par_cyt_	0.40	10.00	3.10
Par_vac_	0.45	2.20	1.00
Dd2			
iRBC_cyt_	0.08	41.00	3.50
Par_cyt_	0.35	8.50	17.80
Par_vac_	0.45	5.20	39.00

### Fluo-4 concentration accumulation in the intact host–parasite system

The greater Fluo-4 fluorescence values seen for Dd2 parasites would suggest that the DV of this strain contains high concentrations of Ca^2+^ (Table 2). This is misleading and was proven to be incorrect by using more reliable ratiometric Fura-Red recordings, which delivered actual steady-state Ca^2+^ concentrations well below the measured Fluo-4 values (Table 2) [12]. As the quantified [Ca^2+^]_free_ represent steady-state values and PfMDR1 actively pumps Fluo-4 into the DV of Dd2 parasites, the pump rate of this transporter cannot be deduced from these values. Furthermore, despite the fact that the bulk concentration of dye is fixed under our conditions, each compartment does not necessarily equilibrate to this dye-level value in the steady-state, in particular not the DV where dye is actively concentrated. Therefore, to deduce the kinetics properties of PfMDR1 on the DV membranes of Dd2 parasites, the measured time course of Fluo-4 fluorescence uptake must be converted into an absolute dye concentration using an equation that also takes into account the differences in pH values between the parasite's cytosol (pH 7.2) and DV (pH 5.2).

**Fig. 5:**
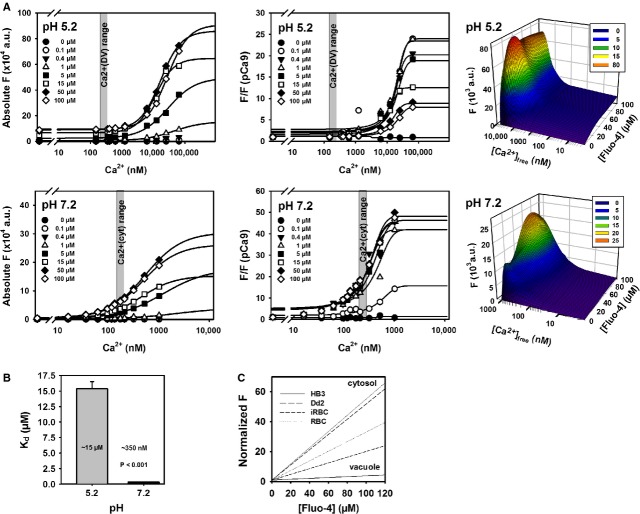
*In vitro* calibration of Fluo-4 fluorescence at varying pH. (**A**) *In vitro* calibration aimed to correlate Fluo-4 concentrations with Fluo-4 fluorescence at given calculated free [Ca^2+^] in a cuvette for vacuolar (5.2) and cytoplasmic (7.2) pH. For each bulk [Fluo-4], a sigmoidal curve was fitted to the data, yielding *K*_*d*_ values for the Fluo-4:Ca^2+^ binding. The expected free steady-state [Ca^2+^] region for the cytoplasm and DV is highlighted in grey. (**B**). Calculated average pooled *K*_*d*_ from the fitted curves to the normalized data in (**A**). The binding affinity is approximately two orders of magnitude lower at vacuolar over cytoplasmic pH (indicated by the greater *K*_*d*_ values at pH 5.2). (**C**) Using the *K*_*d*_ values from (**B**) and the fixed steady-state [Ca^2+^] in the respective compartments (Table 2), a [Fluo-4]-normalized fluorescence conversion curve was constructed to convert F/F(0) values from (Fig. 2) into absolute [Fluo-4].

The fluorescence-Ca^2+^ projections from the fluorescence-[Ca^2+^]_free_-[Fluo-4]_tot_ landscape of the *in vitro* calibration is shown in Figure 5A. Fluorescence projections for selected total [Fluo-4] values at pH 5.2 and 7.2 from these landscapes (both absolute fluorescence values and normalized to background values are shown) are depicted in the left panels of Figure 5A. Sigmoidal fits were used to extract the *K*_*d*_ values of the Fluo-4–Ca^2+^ buffer reaction for all [Fluo-4]_tot_ investigated at each pH (Fig. 5A). As the *K*_*d*_ values do not depend on total Fluo-4 concentration in a closed system (the equilibrium at any given fixed Ca^2+^ concentrations, Eq. (1), just shifts towards larger fluorescence values for larger total dye concentrations, Fig. 5A), the experimental *K*_*d*_ values were averaged together for all [Ca^2+^] at pH 5.2 and 7.2, respectively, as they were not systematically different within each pH value (data not shown). The evaluated *K*_*d*_ values for the Ca^2+^–Fluo-4 system indicate a roughly 40 times lower affinity for Fluo-4 Ca^2+^ binding at pH 5.2 (Fig. 5B).

**Fig. 6:**
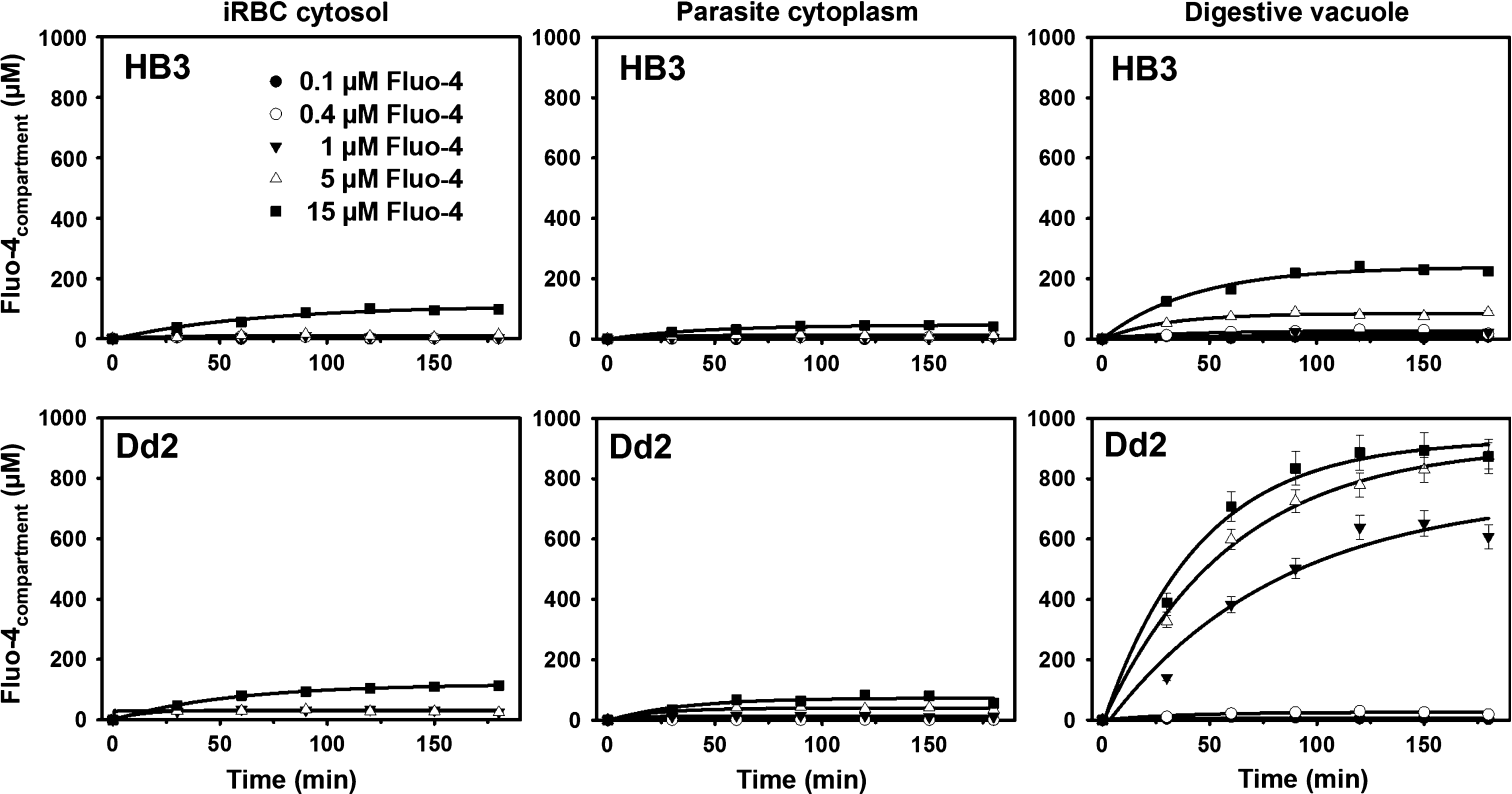
Fluo-4 uptake for varying [Fluo-4]_bulk_. iRBC cytosol, parasite cytoplasm and DV Fluo-4 concentrations during loading with given bulk Fluo-4 AM concentrations. The curves show almost no difference in the iRBC and parasite cytoplasm compartments between HB3 and Dd2 parasites. Uptakes are markedly increased in the DV of Dd2 over HB3 parasites.

Using the known steady-state free Ca^2+^ levels ([Ca^2+^]_free_) from past Fura-Red studies (Table 2) and the *K*_*d*_ values at the pH values of interest (5.2 for DV, 7.2 for parasite cytoplasm and iRBC cytosol), Eq. (1) was used to construct a linear calibration curve relating normalized Fluo-4 fluorescence F/F(0) intensities to an absolute total Fluo-4 concentration [Fluo-4] (Fig. 5C). With this calibration, the fluorescence uptake results from Figure 2 were converted to absolute total compartmental [Fluo-4] concentration ([Fluo-4]_compartment_) curves (Fig. 6).

### Fluo-4 uptake kinetics

**Fig. 7:**
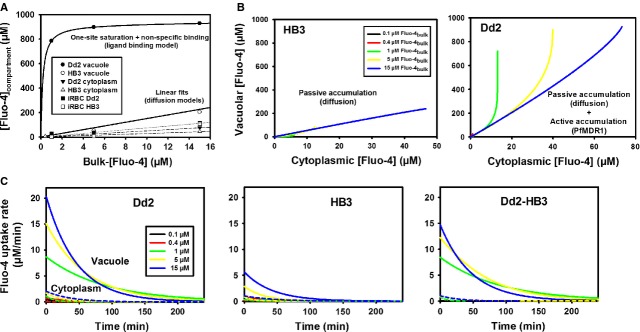
Flux model and pump rates for PfMDR1. (**A**) steady-state (180 min.) compartmental [Fluo-4]_compartment_ *versus* [Fluo-4]_bulk_ relationships reveal linear uptake models for all parasite compartments except for the DV of Dd2 parasites, where the data were best fitted with a ligand-binding model representing the PfMDR1 pump. (**B**) From the compartment uptake curves of [Fluo-4] in Figure 6, the parasitic cytoplasm–DV relationship was reconstructed, which represents the driving force for Fluo-4 from the cytoplasm into the DV. For HB3, this process is entirely compatible with diffusion while for Dd2, diffusion would only allow for a limited vacuolar uptake that is vastly exceeded by the active pump. (**C**) By differentiating the time courses of the uptake fits from Figure 6, Fluo-4 uptake rates are acquired and plotted for the cytoplasm and DV of Dd2 and HB3 parasites, respectively. The plot Dd2-HB3 (far right panel) shows diminished cytoplasmic rates while providing only uptake kinetics *via* the PfMDR1 pump.

Using the kinetics time course data (Fig. 6), we sought to construct a dose–response curve for the relationship of external total [Fluo-4]_bulk_ and internal total [Fluo-4]_compartment_ for each compartment using the steady-state values (Fig. 7A). All compartments, except the DV of Dd2, demonstrate a linear relationship. The ‘Dd2 bulk Fluo-4 input’ to ‘vacuolar Fluo-4 concentration’ relationship (Bulk-[Fluo-4] − [Fluo-4]_DV_) fitted well with a ligand-binding model containing a one-site saturation plus a non-specific binding site of the equation


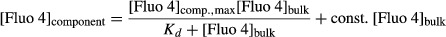
(2)

To express the relationship between the parasitic [Fluo-4] in the cytoplasm and the DV of Dd2 and HB3-infected erythrocytes, the corresponding [Fluo-4] values from both compartments at corresponding time-points from Figure 6 were plotted (Fig. 7B). For HB3 parasites, the vacuolar–cytoplasmic [Fluo-4] relationship is linear for all bulk concentrations used and is compatible with a passive accumulation of Fluo-4 in the HB3 DV (diffusion model). For Dd2, cytoplasmic [Fluo-4] reached greater values compared to HB3 cytoplasm. Moreover, for a given cut-off value for cytoplasmic [Fluo-4] in Dd2 parasites, vacuolar [Fluo-4] bent off to reach a several-fold larger concentration because of active accumulation of Fluo-4 *via* PfMDR1 (Fig. 7B).

To assess the *in situ* uptake rates of Fluo-4, the time course curves of Figure 6 were differentiated in time for the Dd2 and HB3 compartments (Fig. 7C). As Dd2 curves comprise the combined contribution of passive and active vacuolar solute transport, the difference in kinetics (Dd2 − HB3) provides the active transport rate of the PfMDR1 pumps, assuming a similar passive distribution in both HB3 and Dd2 parasites (Fig. 7C, Dd2 − HB3). The vacuolar curves describe exponentially decaying PfMDR1 pump rates for Fluo-4 uptake *in situ* that have concentration-dependent maximum values between 8 and 15 μM/min. for the bulk Fluo-4 concentrations of 1–15 μM tested. From the final vacuolar concentrations (Fig. 6) and the morphometric analysis of the compartmental volumes (Table 3), we can estimate the number of Fluo-4 molecules in the steady-state to be in the order of 3 Mio in the DV of Dd2 parasites with a maximum overall pump rate of ∼50,000 Fluo-4 molecules/min. or 820/sec. for all PfMDR1 molecules present on the DV membrane.

**Table 3:** Volumes of various host–parasite compartments

	Dd2 fl ± SEM (*n*)	HB3 fl ± SEM (*n*)
Digestive vacuole	5.5 ± 0.6 (4)	5.1 ± 0.6 (4)
Parasite cytoplasm	36.3 ± 5.2 (4)	40.8 ± 2.7 (4)
iRBC	130.4 ± 5.7 (4)	113.2 ± 9.3 (4)

## Discussion

It is well known that drug resistance in *P. falciparum* is related to PfMDR1 activity in the membrane of the DV as an ATP-consuming process, however, direct estimates of the pump rate of this transport process in the natural environment of the intact host–parasite system have never been assessed. As previous studies have shown that Fluo-4 is a substrate for PfMDR1 [6,12], we sought to design an approach to use Fluo-4 fluorescence uptake as a measure of compartmental Fluo-4 concentration accumulation in the different compartments of the host–parasite system during dye loading. We performed a ‘reverse imaging’ approach and successfully calculated the rate of transport for PfMDR1 in Dd2 parasites using a ligand-binding model containing one-site saturation and non-specific binding.

The transport of Fluo-4 is mainly influenced by distinct amino acid residues—and not copy number—of the PfMDR1 transporter [6]. We evaluated *pfmdr1* copy numbers for the HB3 and Dd2 parasite strains cultured in our laboratory and found them to have 1 and 2 copies, respectively. To verify for PfMDR1 polymorphisms, we sequenced the complete *pfmdr1* gene of HB3 and Dd2 parasites to ensure that no additional mutations were present that may influence substrate transport. We confirmed three single nucleotide polymorphisms in the two strains at positions 86, 184 and 1042. While HB3 parasites contained N86, F184, D1042 polymorphisms, Dd2 parasites had F/Y86, Y184, N1042. Dd2 parasites revealed an additional variation at position 86, in which one of the two copies of *pfmdr1* contained the amino acid F86 and the other Y86. PfMDR1 mutations at position 86 have been suggested to play a significant role in multidrug resistance [18–20], although numerous studies have also shown that positions 184, 1034, 1042 and 1246 could also be important [15,21,22,24]. Our data propose that polymorphisms at this position are important for the transport of Fluo-4 into the DV of Dd2 parasites. HB3 parasites do not transport Fluo-4 into the DV and seem to only accumulate the dye by passive diffusion.

To better understand the role of PfMDR1 in multidrug resistance, a more comprehensive biochemical characterization (*i.e*. pump rates and kinetics) of this transporter is required. Rather than expressing PfMDR1 in an artificial expression system, which could introduce artefacts to its mode of action, our approach deduces PfMDR1 pump rates directly from intact host–parasite Fluo-4 fluorescence uptake data. Calculating the overall transport rate of PfMDR1 required the conversion of Fluo-4 uptake fluorescence into absolute Fluo-4 concentrations. For this, several parameters were quantified. First, the apparent steady-state *K*_*d*_ was calculated in the respective compartments of live parasites, as these values are profoundly influenced by environmental parameters such as pH, temperature and ionic strength [12]. This must be taken into account when interpreting Fluo-4 fluorescence data in live cells, otherwise the conclusions drawn from the resulting data could be misconstrued. For example, in the past, the DV was suggested to serve as a Ca^2+^ store [25]. This conclusion, however, is incorrect as the above mentioned variables were not applied to the measured Fluo-4 fluorescence. When ratiometric Ca^2+^ dyes were used, it was shown that the DV is not a Ca^2+^ store [12]. Nevertheless, Fluo-4 can be used for live-cell imaging of parasites in another way, as it is a substrate for PfMDR1 and can serve as a valuable asset for monitoring PfMDR1 pump activity through time-lapse recordings of Fluo-4 fluorescence intensities during dye loading protocols. For this approach, steady-state free [Ca^2+^] were assumed as fixed for each compartment and taken from our previous work, where we used Fura-Red to measure resting Ca^2+^ concentrations in Dd2 and HB3 parasites [12]. The calibration of measured Fluo-4 fluorescence with fixed pH (*i.e*. 5.2 and 7.2) and varying known free Ca^2+^ concentrations was required to extract the absolute and normalized *K*_*d*_ values for all Fluo-4 concentrations evaluated at pH 5.2 and 7.2 in this study. With this information, absolute Fluo-4 concentrations for the various compartments of the host–parasite system were obtained for the first time.

Dose–response curves of internal [Fluo-4]_compartment_ at given bulk Fluo-4 concentrations demonstrate that at 1 μM external Fluo-4, the vacuolar Fluo-4 concentration is already saturated in the steady-state between 900 μM and 1 mM. The resulting uptake curves are virtually the same in the iRBC cytosol and the parasite cytoplasm for both HB3- and Dd2-infected erythrocytes. The major difference lies in the vacuolar uptake, which is greatly increased in the DV of Dd2 parasites and reaches extrapolated steady-state values close to ∼1 mM Fluo-4 for the larger bulk concentrations of 5 and 15 μM after ∼200 min. Despite the similar uptake kinetics in the outer compartments, iRBC uptake is nevertheless greater compared to uninfected erythrocytes, which can be explained by the larger drain of Fluo-4 into the parasite compartments of iRBCs.

The vacuolar difference curves of Dd2 − HB3, reflecting the pure PfMDR1 pump behaviour, describe exponentially decaying PfMDR1 pump rates for Fluo-4 uptake *in situ* with concentration-dependent maximum values between 8 and 15 μM/min. for the tested bulk Fluo-4 concentrations of 1–15 μM. This decline in pump rate under *in situ* conditions is expected, as ongoing pump activity fills up the DV with Fluo-4 molecules, which in turn slows down further Fluo-4 uptake. From the final vacuolar concentrations and the morphometric analysis of the compartmental volumes (Table 3), we can estimate the number of steady-state Fluo-4 molecules to be in the order of 3 million in the DV of Dd2 parasites with a maximum pump rate of ∼50,000 Fluo-4 molecules/min.—or 820/sec.—across the DV membrane as a whole. Although it would be good to have the actual pump rate per molecule of PfMDR1, quantitative assessments of the exact expression density of transport molecules have never been obtained. Without knowing the pump rate per molecule of PfMDR1, the conclusions drawn from our current study can only reflect overall pump rate of PfMDR1 in the DV of Dd2 parasites. This must be taken into consideration when comparing different drug-sensitive strains using our methodology in future studies, as differences in overall pump rates may not only relate to differences in *pfmdr1* copy numbers but also to differential PfMDR1 expression density on the DV membrane. We are addressing this issue in future studies using super-resolution microscopy in conjunction with fluorescently tagged PfMDR1.

In conclusion, this is the first direct quantification of *in situ* overall DV pump rates for PfMDR1 in the intact host–parasite system. We were able to determine the rate of solute transport across the DV membrane of Dd2 parasites with the PfMDR1 mutations F/Y86, Y184, S1034, N1042, and D1246. This assay has provided us with a powerful tool to measure the effect of PfMDR1 mutations and copy numbers on substrate (Fluo-4) import kinetics. The methodology can be implemented to monitor drug resistance associated with distinct mutations in the transporter and will be used to screen small molecule libraries available in our laboratories. We are now able to identify which PfMDR1 amino acid mutations can more effectively modulate the transport of any given drug or substrate into the DV. In turn, this will allow for a better understanding of the transporter's mode of action and possibly reveal its natural substrates to us.
